# Combined endoscopic mAnagement of BiliaRy and gastrIc OutLET obstruction (CABRIOLET Study): A multicenter retrospective analysis

**DOI:** 10.1002/deo2.132

**Published:** 2022-06-14

**Authors:** Giuseppe Vanella, Michiel Bronswijk, Roy LJ van Wanrooij, Giuseppe Dell'Anna, Wim Laleman, Hannah van Malenstein, Rogier P Voermans, Paul Fockens, Schalk Van der Merwe, Paolo Giorgio Arcidiacono

**Affiliations:** ^1^ Pancreatobiliary Endoscopy and Endosonography Division, Pancreas Translational and Clinical Research Centre IRCCS San Raffaele Scientific Institute and University Milan Italy; ^2^ Department of Gastroenterology and Hepatology University Hospitals Gasthuisberg University of Leuven Leuven Belgium; ^3^ Department of Gastroenterology and Hepatology Imelda General Hospital Bonheiden Belgium; ^4^ Department of Gastroenterology and Hepatology Amsterdam UMC, Vrije Universiteit, Amsterdam Gastroenterology Endocrinology and Metabolism Amsterdam the Netherlands; ^5^ Department of Gastroenterology and Hepatology Amsterdam UMC, University of Amsterdam, Amsterdam Gastroenterology Endocrinology and Metabolism Amsterdam the Netherlands

**Keywords:** biliary obstruction, endosonography, gastric outlet obstruction, stents, therapeutic endoscopic ultrasonography

## Abstract

**Objectives:**

Combined biliary obstruction and gastric outlet obstruction (GOO) represent a challenging clinical scenario despite developments in therapeutic endoscopic ultrasonography (EUS) as GOO might impair EUS‐guided biliary drainage. Little is known about the effectiveness of different therapeutic combinations used to treat double obstruction, especially regarding stent patency.

**Methods:**

All consecutive patients with double obstruction treated between 2016 and 2021 in three tertiary academic centres were eligible for inclusion. Five combinations involving enteral stenting (ES), EUS‐guided gastroenterostomy (EUS‐GE), hepaticogastrostomy (EUS‐HGS), choledochoduodenostomy (EUS‐CDS), and transpapillary biliary stenting (TPS) were evaluated for dysfunction during follow‐up, either as proportions or dysfunction‐free survival (DFS) using Kaplan–Meier estimates.

**Results:**

Ninety‐three patients were included (male 46%; age 67 [interquartile range 60–76] years; pancreatic cancer 73%, metastatic 57%), resulting in 103 procedure combinations. Different combinations showed significantly different overall dysfunction rates (*p* = 0.009), ranging from the null rate of EUS‐GE+HG to the 18% rate of EUS‐GE+TPS, 31% of EUS‐GE+EUS‐CD, 53% of ES+TPS and 83% of ES+EUS‐CDS. Sub‐analyses restricted to biliary dysfunction confirmed these trends. A multivariate Cox proportional‐hazards regression of DFS, a stenosis distal to the papilla (HR 3.2 [1.5–6.9]) and ES+EUS‐CDS (HR 5.6 [2–15.7]) independently predicted dysfunction.

**Conclusions:**

Despite a lack of statistical power per combination, this study introduces new associations beyond the increased risk of GOO recurrence with ES versus EUS‐GE. EUS‐CDS showed reduced effectiveness and frequent dysfunction in the context of GOO, especially when combined with ES. EUS‐GE+HGS or EUS‐GE+TPS in this setting might result in superior patency. These results suggest that a prospective evaluation of the optimal endoscopic approach to malignant double obstruction is needed.

## INTRODUCTION

Gastrointestinal malignancies represent a frequent cause of cancer‐related mortality.^1^ Jaundice and gastric outlet obstruction (GOO) represent the most frequent complications associated with pancreatobiliary tumors^2^; minimally‐invasive palliation of GOO and obstructive jaundice is a prerequisite for the restoration of quality of life and the initiation/resumption of chemotherapy where applicable.

Whereas endoscopic retrograde cholangiopancreatography (ERCP) with metal stenting remains the gold standard for biliary drainage, this may fail more commonly in the setting of GOO.^3^, ^4^ Endoscopic ultrasonography (EUS)‐guided biliary drainage (EUS‐BD) is considered a valuable alternative in this scenario.^5^ Despite this, little data are available comparing the outcomes of the different EUS‐BD procedures (EUS‐guided choledochoduodenostomy [EUS‐CDS], hepatico‐gastrostomy [EUS‐HGS], EUS‐guided rendezvous, or antegrade stenting), with local expertise and preference often driving treatment selection. As for GOO, growing evidence supports EUS‐guided gastroenterostomy (EUS‐GE) over enteral metal stents (ES) in terms of clinical success and symptoms recurrence.^6^, ^7^, ^8^

Double obstruction represents an additional challenge, as GOO might preclude some therapeutic options and may affect the outcome of successfully completed biliary drainage. Despite this potential interplay, the combined management of double obstruction has not been deeply analyzed, with only a small series describing specific combinations.^9^, ^10^, ^11^, ^12^

The aim of this study was to analyze and compare the outcomes of different procedure combinations aimed at treating double obstruction.

## METHODS

This is a retrospective evaluation of prospectively maintained databases of three tertiary, academic, referral centres: San Raffaele Hospital (Milan, Italy), University Hospitals UZ Leuven (Belgium), and Amsterdam UMC (the Netherlands). All consecutive procedures aimed at treating a double obstruction between January 2016 and October 2021 were included.

### Aim

The primary aim was to analyze the dysfunction of different therapeutic combinations in the management of double obstruction, both as rate (proportion) and time‐to‐event (dysfunction‐free survival [DFS]). Secondary outcomes were the rates of technical success, clinical success, and adverse events (AEs).

### Definitions and patients inclusion

All included patients had a histologically confirmed malignancy.

In the presence of a radiologically or endoscopically confirmed biliary and upper gastrointestinal malignant stenosis, biliary obstruction (BO) was defined as the presence of jaundice (bilirubin ≥2 mg/dl), whereas GOO was defined as a GOO Scoring System (GOOSS^13^) <2 (no intake or liquids only),

Double obstruction was defined as the combined presence of BO and GOO. An interval <180 days was allowed between biliary and alimentary procedures. A minimal post‐procedural follow‐up of 30 days was required unless death occurred earlier.

The level of duodenal stenosis was defined as: type I: proximal to the major papilla; type II: involving the major papilla; and type III: distal to the major papilla.

The following procedure combinations were performed:

(a) ES+trans‐papillary self‐expandable metal stent (TPS)
(b) ES+EUS‐CDS
(c) ES+EUS‐HGS
(d) EUS‐GE+TPS
(e) EUS‐GE+EUS‐CDS
(f) EUS‐GE+EUS‐HGS

All TPS were included independently of the route through which they were placed (ERCP‐, percutaneous‐, or EUS‐guided).

Technical success (TS) was defined as the completion of the intended procedure. Among technically successful procedures, clinical success (CS) was defined as a postprocedural >50% bilirubin reduction for BO and a postprocedural GOOSS ≥ 2 corresponding to the possibility to tolerate a soft solid diet for GOO.

AEs were scored through the ASGE lexicon as mild, moderate, severe, or fatal.^14^

Dysfunction was defined as the recurrence of either jaundice or GOO after a former clinical success. DFS was defined as the time from the completion of a specific combination to the first dysfunction (either biliary or alimentary).

As for the analysis of combinations, combined TS and CS required both procedures to be completed and successful, whereas an AE or a dysfunction in either of the two procedures was sufficient for assigning an AE or dysfunction to that combination.

### Interventional EUS procedures

All procedures were performed under deep sedation or general anaesthesia, in a fluoroscopy‐equipped room, and using linear echoendoscopes (EG34‐J10U; Pentax Medical). EUS‐GE was performed using the wireless simplified EUS‐GE technique,^15^ involving an oro‐jejunal tube for jejunal distension and free‐hand placement of an electrocautery‐enhanced 20 or 15 mm lumen‐apposing metal stent (LAMS; Hot Axios; Boston Scientific).^16^ EUS‐CDS was performed through the free‐hand placement of an 8 × 8 or a 6 × 8‐mm LAMS between the common bile duct and the duodenum. EUS‐guided intrahepatic access was performed by a 19‐G needle and guidewire cannulation (0.025‐inch Visyglide; Olympus or 0.035‐inch Jagwire; Boston Scientific). The tract was created through a 6‐Fr cystotome (Endoflex). In the case of EUS‐HGS, a partially covered stent (Giobor, Taewoong) was placed.^17^

### Ethics

This study was conducted in compliance with the Declaration of Helsinki and Good Clinical Practice. The protocol was approved by the Ethics Committee at the coordinating centre (Id: 178/INT/2020) and each location.

### Statistics

Descriptive statistics are reported as frequencies (proportions) and medians (interquartile ranges).

Comparisons were performed through the Chi‐squared or Fisher's test for qualitative data, and the Mann–Whitney or Kruskal–Wallis test for quantitative data.

Dysfunction‐free survival was analyzed by Kaplan–Meier statistics, with a log‐rank test for comparison between subgroups. Patients were censored when experiencing dysfunction or on the last day of follow‐up or death, whichever came first. A stepwise Cox proportional‐hazards regression was performed and results were expressed as hazard ratio (HR) and a 95% confidence interval.

A *p*‐value <0.05 was considered significant. All analyses were performed using Medcalc (Ostende, Belgium).

### Sub‐analyses

As a significantly higher GOO recurrence is expected in the ES group versus the EUS‐GE group independently from any combination,^8^ a subgroup analysis was performed analysing only biliary events (biliary vs. no dysfunction), excluding all patients experiencing GOO recurrence.

For the same reason, a subgroup analysis was performed including only patients in which GOO was treated by EUS‐GE.

## RESULTS

Between 2016 and 2021, 93 patients fulfilling inclusion criteria were treated for double obstruction in the participating centers (Table 1). The median age was 67 [60–76] years, and 46.2% were male. The primary disease was pancreatic cancer in 73% and duodenal/ampullary cancer in 11%. The disease stage was metastatic in 57% of cases, and 20% with the peritoneal disease. Jaundice was the first presenting symptom in 65%, GOO in 9%, while they were concomitant in 26% of patients.

**TABLE 1:** Characteristics of included patients

Variable	Total (*N* = 93)
Age, median [IQR]	67 [60–76]
Male, *n* (%)	43 (46.2%)
Primary disease	
Pancreatic cancer	68 (73.1%)
Duodenal / ampullary cancer	10 (10.7%)
Cholangiocarcinoma	4 (4.3%)
Others*	11 (11.8%)
Oncological staging	
Resectable	4 (4.3%)
Borderline resectable	5 (5.4%)
Locally advanced	29 (31.2%)
Metastatic	53 (57%)
N.A.	2
Ascites	18 (19.4%)
Peritoneal disease	19 (20.4%)
Symptoms onset	
Biliary first	60 (64.5%)
Concomitant	24 (25.8%)
Gastric outlet obstruction first	9 (9.7%)
Median interval between procedures, days [IQR]	31 [5‐68]

Abbreviation: N.A., not available.

* Gallbladder cancer/neuroendocrine tumors/metastatic diseases.

These 93 patients resulted in 103 procedure combinations. The median interval between alimentary and biliary procedures was 31 [5–68] days

Characteristics of biliary and GOO procedures are analyzed in Tables S1 and S2 reporting relative efficacy/safety data for each technique.

### Procedure combinations

**FIGURE 1:**
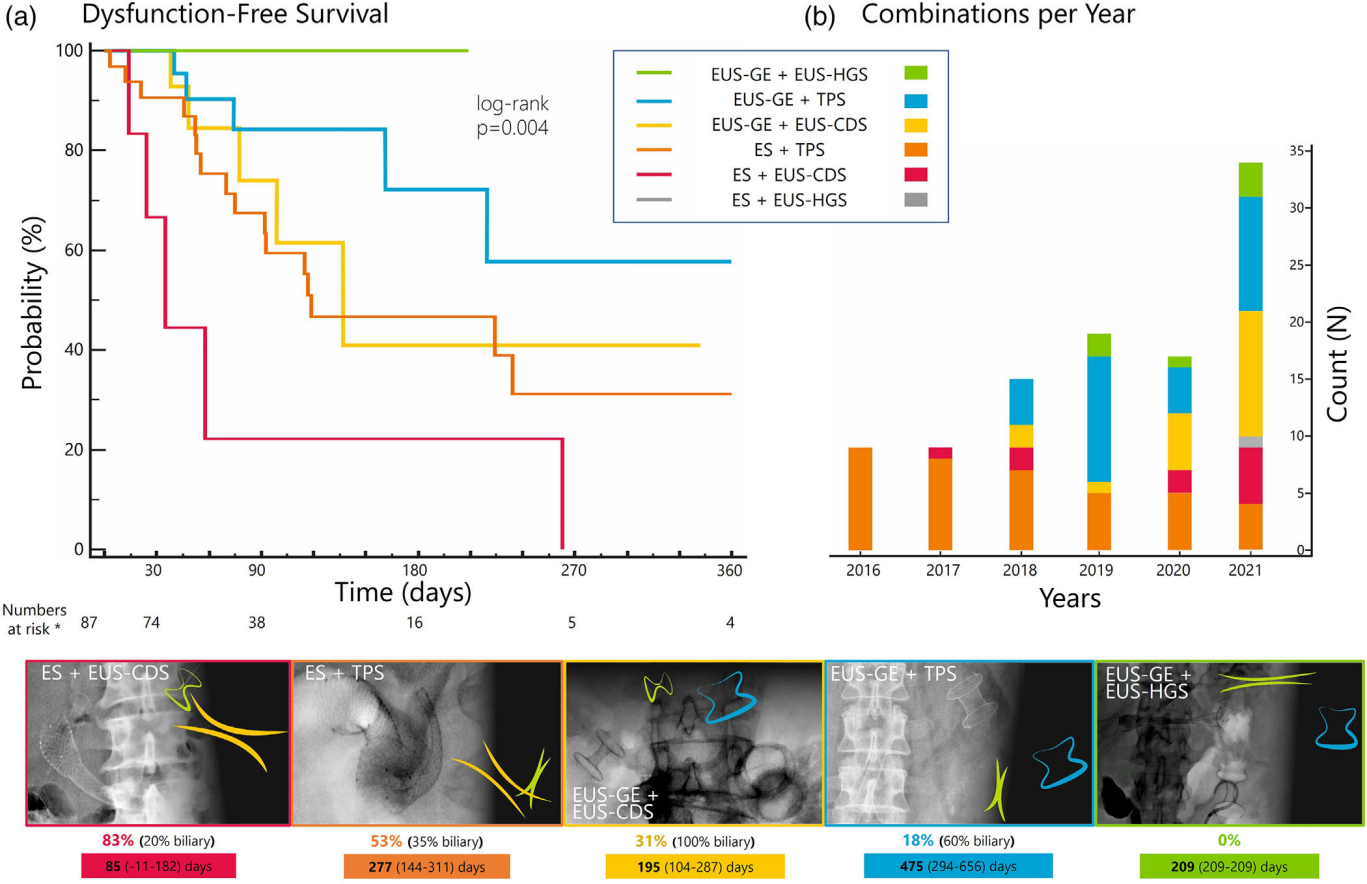
Different combinations used to treat double obstruction during the study period. (a) Dysfunction‐free survival probability of different combinations at Kaplan–Meier analysis. (b) Number of cases per year (columns) with different colors within columns representing the number of specific combinations. Charts: Fluoroscopic images and schematic representation of all different combinations included in this protocol; below each combination, the overall dysfunction rate (with the relative rate of biliary vs. GOO recurrence) and the mean estimated dysfunction‐free‐survival (days) from Kaplan‐Meier analysis are provided. ^*^Numbers at risk per each combination: ES+TPS = 32; ES+EUS‐CDS = 6; EUS‐GE+TPS = 28; EUS‐GE+CDS = 16; EUS‐GE+HGS = 5

Endoscopic management of double obstruction increased from nine cases/year in 2016/2017 to 34 cases/year in 2021 (Figure 1b). There was a significantly different distribution of combinations along the years (Figure 1b), with a relative increase of EUS‐GE combinations, especially EUS‐GE+EUS‐CDS, and a relative decrease of ES+TPS (*p* < 0.001).

Outcomes of different combinations are described in Table 2.

**TABLE 2:** Outcomes of different therapeutic combinations

Variable	ES+TPS (*N* = 38)	ES+EUS‐CDS (*N* = 10)	ES+EUS‐HGS (*N* = 1)	EUS‐GE+TPS (*N* = 29)	EUS‐GE+EUS‐CD (*N* = 19)	EUS‐GE+EUS‐HGS (*N* = 6)	*p*‐value
Combined technical success	38	10	0	29	18	6	
Combined clinical success	32/38 (84.2%)	6/10 (60%)		28/29 (96.6%)	16/18 (88.9%)	5/6 (83.3%)	0.07
*GOO*	84%	70%		97%	89%	100%	
*Biliary*	100%	80%		100%	100%	83%	
Combined AEs	7 (18.4%)	2 (20%)	1 (100%)	7 (24.1%)	5 (26.3%)	1 (16.7%)	0.5
Combined recurrences	17/32 (53.1%)	5/6 (83.3%)		5/28 (17.9%)	5/16 (31.2%)	0/5 (0%)	0.002
*GOO versus biliary*	65% vs. 35%	80% vs. 20%		40% vs. 60%	0% vs. 100%	0	
Median FU, days [IQR]	93 [44–156]	33 [24–58]	/	77 [38–158]	74 [44–105]	37 [30–110]	0.3
*Kaplan‐Meier analyses*							
Mean estimated symptoms‐free survival (95% CI), days	277 (CI 144–311)	85 (CI 11–182)		475 (CI 294–656)	195 (CI 104–287)	209 (CI 209–209)	Log‐rank *p* = 0.004
DFS probability							
30 days	90.6%	66.7%		100%	100%	100%	
3 months	67.4%	22.2%		84.2%	73.9%	100%	
6 months	46.7%	22.2%		72.2%	41%	100%	
1 year	31.2%	0%		57.7%	41%	100%	

Abbreviations: AEs, adverse events; DFS, dysfunction‐free survival; ES, enteral stent; EUS‐CDS, EUS‐guided choledochoduodenostomy; EUS‐GE, EUS‐guided gastroenterostomy; EUS‐HGS, EUS‐guided hepaticogastrostomy; GOO, gastric outlet obstruction; TPS, transpapillary self‐expandable metal stent.

#### Clinical success

**FIGURE 2:**
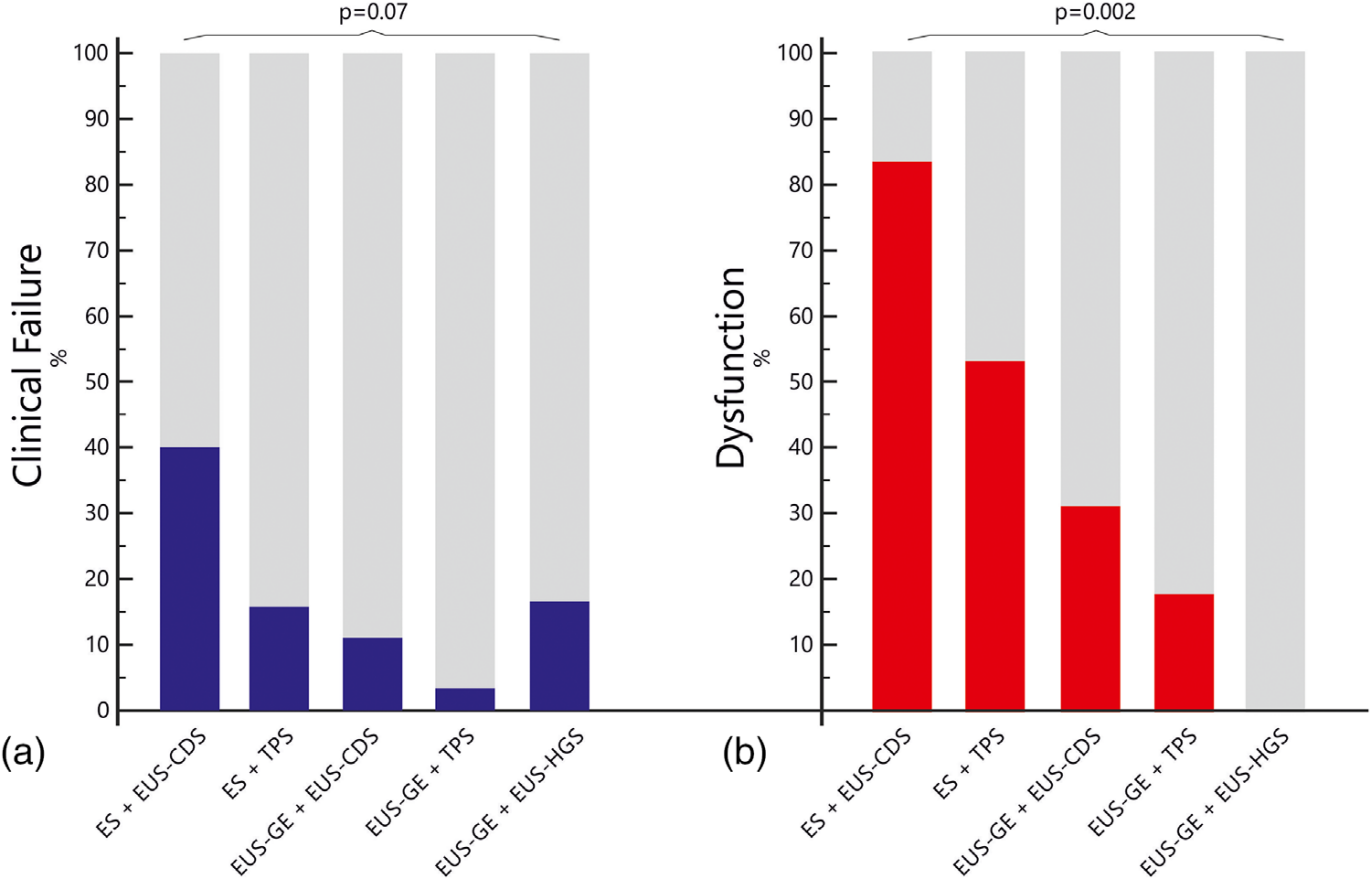
Frequencies bar chart showing the relative rate of (a) clinical failure and (b) dysfunction per each combination

A higher primary failure of ES+EUS‐CDS was noted (40%) with respect to other combinations (11%, *p* = 0.02); see Figure 2a.

#### Dysfunction

ES in combination with EUS‐CDS resulted in a significantly higher overall dysfunction (83%). Overall dysfunction rate was 53% with ES+TPS, 31% with EUS‐GE+EUS‐CDS, 18% with EUS‐GE+TPS, and 0 with EUS‐GE+EUS‐HGS (*p* = 0.002; Table 2 and Figure 2b)

Through Kaplan–Meier statistics (see Figure 1a), these events resulted into a 6‐month probability of DFS of 22%, 47%, 41%, 72%, and 100%, respectively (log‐rank *p* = 0.004), together with different estimated mean time‐to‐dysfunction (Table 2 and Figure 1).

### Biliary events only

After excluding GOO recurrences, 70 clinically successful combinations remained in the analysis (see Table S3). Despite no statistical significance, data on biliary dysfunction confirmed the trends observed in the general analysis, with EUS‐GE+EUS‐HGS showing no dysfunction, while the rate was 12% with EUS‐GE+TPS, 28% with ES+TPS, 31% with EUS‐GE+EUS‐CDS, and 50% with ES+EUS‐CDS (*p* = 0.2).

### EUS‐GE only

When including only patients in whom GOO was treated through EUS‐GE, 53 clinically successful combinations remained (see Table S4). Dysfunction data confirmed the trends observed in the general analysis, with EUS‐GE+EUS‐HGS showing no dysfunction, while the rate was 18% in EUS‐GE+TPS and 31% in EUS‐GE+EUS‐CDS.

### Predictors of dysfunction

**FIGURE 3:**
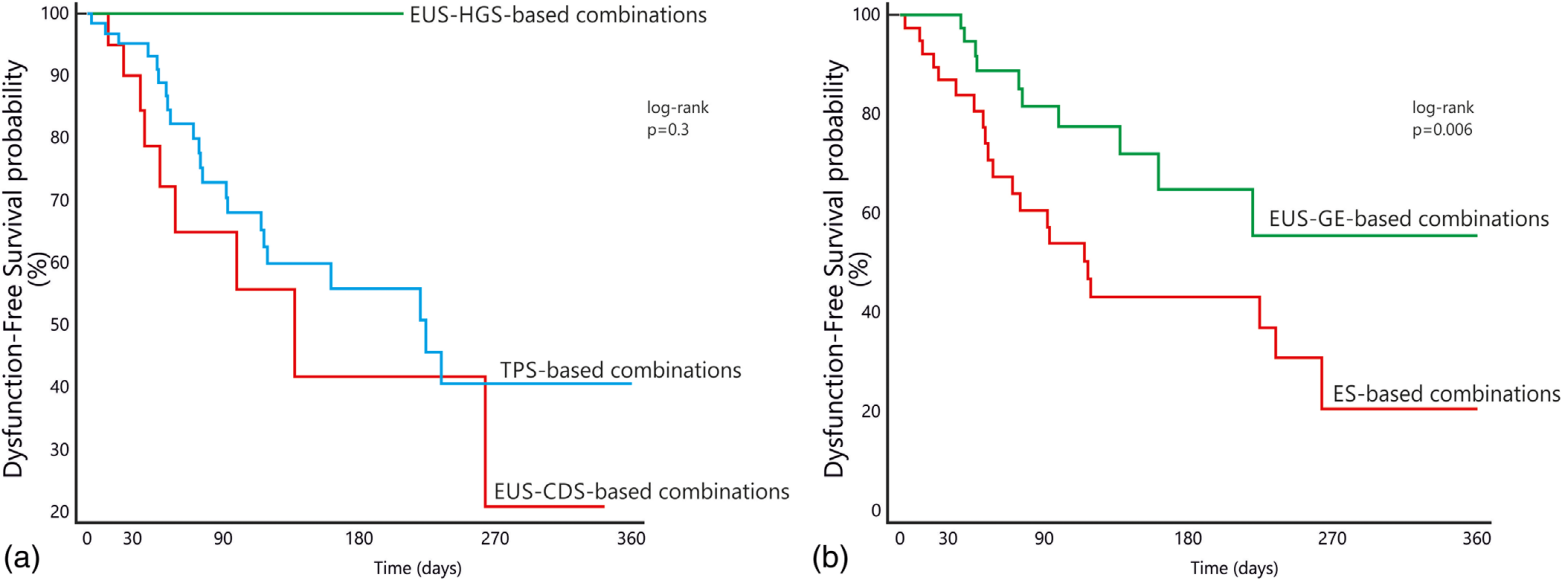
Dysfunction‐free survival probability separated according to (a) the biliary obstruction being treated by EUS‐hepaticogastrostomy (EUS‐HGS), EUS‐choledochoduodenostomy (EUS‐CDS), or transpapillary biliary metal stents (TPS). (b) The gastric outlet obstruction being treated by EUS‐gastroenterostomy (EUS‐GE) versus enteral stenting

When comparing combinations where dysfunction was detected, versus those not resulting in any event during follow‐up (Table 3), no influence of age, sex, underlying disease, or disease stage was found. In univariate analysis, the duodenal stenosis being distal to the papilla increased the probability of any dysfunction (HR 2.6 [1.2–5.4]). No role was found for biliary procedures (Figure 3a), whereas choosing ES for GOO resulted in a lower probability of remaining dysfunction‐free (HR 0.4 [0.2–0.8]; Figure 3b).

**TABLE 3:** Recurrences

Variable	Recurrence (*N* = 32)	No recurrence (*N* = 55)	*p*‐value	Univariate analysis	Multivariate analysis
Age, days [IQR]	66 [62–75]	64 [57–77]	0.6		
Male sex	12 (37.5%)	26 (47.3%)	0.4		
Primary disease			0.4		
Pancreatic cancer	24 (75%)	38 (69.1%)			
Duodenal/ampullary cancer	6 (18.8%)	7 (12.7%)			
Cholangiocarcinoma	0	3 (5.5%)			
Others	2 (6.2%)	7 (12.7%)			
Ascites	3 (9.4%)	12 (21.8%)	0.1		
Carcinomatosis	2 (6.2%)	15 (27.3%)	0.02	NS	
Type of duodenal stenosis			0.005		
Proximal to the papilla	11 (34.4%)	30 (54.5%)		1	
Involving the papilla	10 (31.2%)	21 (38.2%)		NS	
Distal to the papilla	11 (34.2%)	4 (7.3%)		HR 2.6 [1.2–5.4]	HR 3.2 [1.5–6.9]
BIliary management			0.2		
EUS‐CDS	9 (28.1%)	11 (20%)			
EUS‐HGS	0	5 (9.1%)			
TPS	23 (71.9%)	39 (70.9%)			
GOO management			0.0003		
ES	22 (68.7%)	16 (29.1%)		1	
EUS‐GE	10 (31.2%)	39 (70.9%)		HR 0.4 [0.2‐0.8]	
Procedure combination			0.002		
EUS‐GE+EUS‐HGS	0	5 (9.1%)		1	1
EUS‐GE+TPS	5 (15.6%)	23 (41.8%)		NS	NS
EUS‐GE+EUS‐CDS	5 (15.6%)	11 (20%)		NS	NS
ES+TPS	17 (53.1%)	15 (27.3%)		HR 2.3 [1.1–5.2]	NS
ES+EUS‐CDS	5 (15.6%)	1 (1.8%)		HR = 6.5 [2.2–19.2]	HR 5.6 [2–15.7]

Abbreviations: ES, enteral stent; EUS‐CDS, EUS‐guided choledochoduodenostomy; EUS‐HGS, EUS‐guided hepaticogastrostomy; EUS‐GE, EUS‐guided gastroenterostomy; GOO, gastric outlet obstruction; HR, hazard ratio; NS, not significant; TPS, transpapillary self‐expandable metal stent.

Amongst specific procedure combinations, when compared to EUS‐GE+EUS‐HGS (the combination showing no dysfunction), ES+TPS and ES+EUS‐CDS had a significantly higher dysfunction risk (HR 2.3 [1.1–5.2] and 6.5 [2.2–19.2], respectively).

In multivariate analysis, the stenosis being distal to the papilla (HR 3.2 [1.5‐6.9]) and the combination of ES+EUS‐CDS (HR 5.6 [215.7]) were confirmed as independent predictors of dysfunction, whereas the GOO being initially treated by ES versus EUS‐GE was not.

## DISCUSSION

Combined management of biliary and gastric outlet obstruction remains a challenge. The advent of therapeutic EUS has facilitated a multitude of new therapeutic options. However, the most ideal approach to managing a double obstruction remains unclear. In this large retrospective multicenter analysis, almost 60% of patients presented with metastatic disease, whereas 20% exhibited peritoneal disease and ascites, illustrating the oncological context and adverse prognosis of this association. We confirmed that combinations including ES led to higher primary clinical failure and dysfunction^8^; moreover, specific therapeutic combinations strongly influenced the rate of dysfunction over time. Based on a low number of observations, the combination of EUS‐GE+HGS showed the lowest risk, followed by EUS‐GE+TPS; conversely, combinations including EUS‐CDS showed a higher risk, especially when associated with enteral stenting. In addition, malignant gastrointestinal stenosis distal to the major papilla and the combination of ES+EUS‐CDS were identified as independent predictors of dysfunction.

The gold standard for biliary drainage is ERCP, but this may fail more commonly in patients with antroduodenal infiltration.^3^, ^4^, ^18^ EUS may facilitate ERCP by placing a guidewire for rendezvous if the duodenoscope can be advanced beyond the stricture. Biliary drainage can also be established by EUS‐CDS or EUS‐HGS, or EUS‐guided antegrade stenting, avoiding the morbidity associated with percutaneous drainage.^17^, ^19^

Although surgical gastrojejunostomy has shown better long‐term results, ES is still widely used for malignant GOO,^20^ despite providing suboptimal relief from symptoms and a higher risk of recurrence.^8^ EUS‐GE is increasingly being used as an alternative to both ES and surgical bypass, as it avoids the invasiveness of surgery and the risk of primary failure, recurrence, and need for re‐interventions following ES.^8^, ^16^

Selecting the most optimal approach becomes more complicated when biliary and GOO occur together. Some procedure combinations may interfere with each other. For example, an enteral stent may compromise a previously placed TPS or prevent cannulation of the papilla through the meshes.^21^, ^22^ In addition, EUS‐BD will only be successful once GOO has been fully resolved.^23^

EUS has revolutionized the endoscopic management of simultaneous biliary and GOO. However, limited non‐comparative series have mainly evaluated either individual procedures or single specific combinations in double obstruction.^9^, ^10^, ^11^, ^12^ Our study offers some insight on the risk of dysfunction of different combinations used in the management of double obstruction in three tertiary referral centers.

Over the course of 5 years, we found increased endoscopic management of double obstruction, despite the activity reduction due to the COVID‐19 pandemic,^24^, ^25^ probably related to the increased implementation of therapeutic EUS at our institutions.

In our study, different procedural combinations showed significantly different rates of dysfunction. Specifically, ES combined with EUS‐CDS showed the highest, followed by ES+TPS, whereas EUS‐GE+EUS‐HGS showed the lowest risk. We considered the possibility that our results were affected by the higher GOO recurrence risk following ES placement. However, ES was not an independent predictor of dysfunction, whilst some specific combinations were independently associated with a higher probability of dysfunction and shorter DFS.

To date, the largest published experiences on double obstruction involve ES and ERCP. ERCP has shown suboptimal success in 75% of cases (34%–85%)^26^, ^27^, ^28^ in the context of duodenal invasion. In this setting EUS‐BD demonstrated higher technical success than transpapillary stenting (95.2% vs. 56%^29^) without any difference in AEs, as well as lower stent dysfunction (14% vs. 54%^30^). However, most experiences described EUS‐CDS before the implementation of LAMS. In a recent study reporting the management of double obstruction in patients with an indwelling uncovered duodenal stent,^9^ same‐session EUS‐HGS seemed to reduce overall AEs and resulted in longer patency with respect to other biliary drainage modalities such as percutaneous drainage. In another study analysing EUS‐BD in the case of ES, EUS‐CDS was associated with a higher rate of ascending cholangitis and stent dysfunction than EUS‐HGS (median stent patency 43 vs. 133 days; HR 0.5 [0.2–1], *p* = 0.05).^10^ Conversely, a recent series of 23 patients reported a 95.6% successful through‐the‐meshes LAMS placement,^11^ with no jaundice recurrence after 241 (81–387) days; however, 61% of procedures were performed by EUS‐guided gallbladder drainage. Another series described 23 same‐session EUS‐HGS and EUS‐GE with excellent technical success; 72.7% of patients showed a 50% bilirubin reduction, while 100% of patients tolerated a soft diet with a median hospital stay of 2 days. The rate of AEs was 21% (no severe or fatal AE), whilst 13% required biliary re‐interventions.^12^

A recent meta‐analysis suggested that EUS‐CDS reduced the risk of reinterventions compared to EUS‐HGS.^31^ However, our findings suggest that choledochoduodenostomy drainage might be compromised in the context of a double obstruction.

Considering the strengths of our study, we included all different procedural combinations. Apart from confirming a higher reintervention rate for patients treated by ES versus EUS‐GE, our study suggests that EUS‐CDS in the context of GOO may be associated with suboptimal outcomes following placement and during follow‐up. This is especially true when EUS‐CDS is performed in combination with an enteral stent, but also observed when GOO has been resolved by EUS‐GE. A potential explanation could be that the duodenal bulb may function as a “reservoir” for food residue, increasing the risk of cholangitis through the adjacent LAMS.

According to previous literature and the small number of patients receiving EUS‐GE and EUS‐HGS in our study, this combination seems a promising alternative. These procedures are, however, technically demanding, require specific training, and may lead to serious AEs. The generalizability of our results outside tertiary academic referral centers can therefore not be assured.^5^ As EUS‐GE associated with transpapillary stenting showed the second‐lowest dysfunction rate, also TPS (retrograde or antegrade via EUS or percutaneous guidance) seems a valuable alternative according to local expertise.

This study has several limitations, beyond generalizability. First, the retrospective nature might have missed significant events, despite the fact that advanced EUS procedures in our centers are being included in prospective databases. Second, as enteral stents are more prone to GOO recurrence during FU, an analysis restricted to biliary events would have been desirable. However, as the adequate management of GOO has a significant impact on the efficacy of biliary drainage, the exclusion of patients experiencing GOO recurrence and changing management strategy theoretically dilutes the possibility to detect biliary dysfunctions: we, therefore, preferred to report on overall dysfunction. Notwithstanding, sub‐analyses restricted to biliary events and EUS‐GE‐based combinations confirmed the trends of the general analysis, underlining the reliability of observed associations. Third, the number of procedures in each combination is significantly underpowered to draw firm conclusions, and these results should therefore be regarded as exploratory.

Although this is not a comparative study of pre‐planned strategies, and the different combinations reflect evolutive management of double obstruction over the recent years (based on changing expertise, published evidence, and multidisciplinary awareness), the current results have already influenced our actual clinical practice.

EUS‐GE has become the procedure of choice for GOO in our centers.^8^ As for jaundice, if the stenosis is distal to the papilla (type III) retrograde cannulation will be first attempted, a stenosis proximal to or involving the papilla (I or II) might preclude ERCP; in those cases, antegrade stenting or a EUS‐HGS is usually performed depending on the possibility to direct the guidewire transpapillary. Our results have discouraged us from using EUS‐CDS in the context of GOO, even when resolved by EUS‐GE, especially in type III duodenal stenosis. In all cases, EUS‐GE will be performed first, as the success of EUS‐guided biliary drainage depends on resolving the gastric outlet obstruction.^23^

To our best knowledge, this is the largest study exploring the issue of endoscopic management of both biliary and gastric outlet obstruction, the first evaluating and comparing all possible management combinations, and the only reporting on EUS‐GE+EUS‐CDS. While tertiary referral centers are increasingly replacing ES with EUS‐GE, the type of EUS‐BD in the context of GOO should be further explored in well‐designed prospective clinical studies.

## CONFLICT OF INTEREST

Michiel Bronswijk has consultancy agreements with Taewoong/ Prion Medical, and reports travel grants from Taewoong, Norgine, and Prion Medical; Roy LJ van Wanrooij holds a consultancy agreement with Boston‐Scientific. Wim Laleman co‐chairs the Boston‐Scientific Chair in Therapeutic Biliopancreatic Endoscopy, and has consultancy agreements with Boston Scientific and Cook Medical. Hannah van Malenstein holds a consultancy agreement with Boston‐Scientific. Paul Fockens holds a consultancy agreement with Olympus and Cook Medical. Schalk van der Merwe co‐chairs the Boston‐Scientific Chair in Therapeutic Biliopancreatic Endoscopy, holds the Cook Medical chair in Portal Hypertension, and holds consultancy agreements with Boston Scientific, Cook Medical and Pentax. Rogier P Voermans reports a consultancy agreement and research grant from Boston‐Scientific. All other authors declare no conflict of interest.

## FUNDING INFORMATION

None.

## Supporting information


**Supplementary Table 1**: Characteristics of biliary procedures.
**Supplementary Table 2**: Characteristics of gastric outlet procedures
**Supplementary Table 3**: Biliary Events only
**Supplementary Table 4**: EUS‐GE‐based combinations only
