# Supplementary material for: Combined endoscopic mAnagement of BiliaRy and gastrIc OutLET obstruction (CABRIOLET Study): A multicenter retrospective analysis
Source: DEN Open. 2022 Jun 14;3(1):e132. doi: 10.1002/deo2.132 (PMC9307724; doi:10.1002/deo2.132)
Supplement: Supplementary file 1 — Supplementary Table 1: Characteristics of biliary procedures. Supplementary Table 2: Characteristics of gastric outlet procedures Supplementary Table 3: Biliary Events only Supplementary Table 4: EUS‐GE‐based combinations only [file DEO2-3-e132-s001.docx]

### **Supplementary Table 1**: Characteristics of biliary procedures

| **Variable** | **Total N = 103** |
| --- | --- |
| Previous biliary drainage | 24 (25.8%) |
|  | |
| Biliary Procedure |  |
| Transpapillary biliary metal stents (TPS) | 69 (67%) |
| Retrograde TPS (ERCP) | 46 (44.7%) |
| Antegrade TPS (PTBD or EUS-guided) | 18 (17.5%) |
| Through-the-mesh TPS (ERCP) | 5 (4.9%) |
| EUS-guided Choledochoduodenostomy | 27 (26.2%) |
| EUS-guided Hepaticogastrostomy | 7 (6.8%) |
|  | |
| Outcomes |  |
| Technical Success | 102 (99%) |
| *1 EUS-HGS intraprocedural dislocation* |  |
| Clinical Success | 99/102 (97.1%) |
| *25/27 (92.6%) EUS-CDS vs 5/6 (83.3%) EUS-HGS vs 69/69 (100%) TPS* | *p=0.02* |
| Adverse events | 13 (12.6%) |
| *EUS-CDS : 4/27 (14.8%)*   - *2 Cholangites (1 moderate, 1 fatal)* - *1 Pneumoperitoneum (moderate)* - *1 Stent dislocation (mild)*   *EUS-HGS : 2/7 (28.6%)*   - *1 Stent dislocation (severe)* - *1 Sepsis (moderate)*   *TPS : 7/69 (10.1%)*   - *2 pancreatitis (1 mild, 1 moderate)* - *3 cholangites (mild)* - *2 bleedings (1 moderate, 1 severe)* | *p=0.4* |
| *Mild*  *Moderate*  *Severe*  *Fatal* | *5 (38.5%)*  *5 (38.5%)*  *2 (15.4%)*  *1 (7.7%)* |
| ERCP : Endoscopic Retrograde Cholangiopancreatography ; EUS-guided Choledochoduodenostomy ; EUS-HG : EUS-guided Hepaticogastrostomy ; PTBD : Percutaneous Transhepatic Biliary Drainage ; TPS : Transpapillary Self-Expandable Metal Stent; TTM-TPS : Through-the-meshes TPS | |

### **Supplementary Table 2**: Characteristics of Gastric Outlet procedures

| **Variable** | **Total N = 103** |
| --- | --- |
| Previous GOO management | 7 (6.8%) |
| GOOS Score at diagnosis |  |
| 0 (Total obstruction) | 85 (82.5%) |
| 1 (Liquids only) | 15 (14.6%) |
| 2 (Soft Solids only) | 3 (2.9%) |
| Type of duodenal stenosis |  |
| Above the papilla | 50 (48.5%) |
| Involving the papilla | 37 (35.9%) |
| Below the papilla | 16 (15.5%) |
|  | |
| GOO Procedure |  |
| Enteral stenting | 49 (47.6%) |
| Over biliary TPS / Independent from biliary TPS | 23 vs 26 |
| EUS-guided Gastroenterostomy | 54 (52.4%) |
|  | |
| Outcomes |  |
| Technical Success | 102 (99%) |
| *1 EUS-GE misdeployment* |  |
| Clinical Success | 90/102 (88.2%) |
| *40/49 (81.6%) ES vs 50/53 (94.3%) EUS-GE* | *p=0.047* |
| Adverse events | 12 (11.7%) |
| *ES : 8/49 (16.3%)*   - *2 Aspiration pneumonia (2 moderate)* - *2 Cholangites (2 moderate)* - *2 Fever (1 mild, 1 moderate)* - *1 Hematemesis (moderate)* - *1 Abdominal pain (mild)*   *EUS-GE : 4/53 (7.5%)*   - *1 Misdeployment (severe)* - *1 Cholangites (severe)* - *1 Fever (mild)* - *1 Bleeding (moderate)* | *p=0.2* |
| *Mild*  *Moderate*  *Severe* | *3 (25%)*  *7 (58.3%)*  *2 (16.7%)* |
| ES : Enteral Stenting ; EUS-GE : EUS-guided Gastroenterostomy ; GOO : Gastric Outlet Obstruction ; TPS : Transpapillary Self-Expandable Metal Stent | |

### **Supplementary Table 3**: Biliary Events only

| **Variable** | ES+TPS (N=21) | ES+EUS-CDS (N=2) | EUS-GE+TPS (N=26) | EUS-GE+EUS-CDS (N=16) | EUS-GE+EUS-HGS (N=5) | p-value |
| --- | --- | --- | --- | --- | --- | --- |
| Combined AEs | 5 (23.8%) | 0 (0%) | 6 (23.1%) | 2 (12.5%) | 1 (20%) | p=0.8 |
| Combined Biliary Recurrence | 6 (28.6%) | 1 (50%) | 3 (11.5%) | 5 (31.2%) | 0 (0%) | p=0.2 |
| *Kaplan-Meier analyses* |  |  |  |  |  |  |
| Mean estimated Dysfunction-free-Survival (95% CI), days | 363 (CI 254-472) | 263 (CI 263-263) | 526 (CI 337-714) | 195 (CI 104-287) | 209 (CI 209-209) | Log-rank p=0.3 |
| DFS Probability |  |  |  |  |  |  |
| 30 days | 90.5% | 100% | 100% | 100% | 100% |  |
| 3 months | 77.6% | 100% | 94.4% | 74% | 100% |  |
| 6 months | 70.5% | 100% | 81% | 41% | 100% |  |
| 1 year | 70.5% | 0% | 64.8% | 41% | 100% |  |
| Only technically and clinically successful combinations included.  AEs : Adverse Events ; DFS : Dysfunction-Free Survival ; ES : Enteral Stent ; EUS-CDS : EUS-guided Choledochoduodenostomy; EUS-GE : EUS-guided Gastroenterostomy ; EUS-HGS : EUS-guided Hepaticogastrostomy ; TPS : Transpapillary Self-Expandable Metal Stent | | | | | | |

### **Supplementary Table 4**: EUS-GE based combinations only

|  | EUS-GE+TPS (N=29) | EUS-GE+EUS-CDS (N=18) | EUS-GE+EUS-HGS (N=6) | p-value |
| --- | --- | --- | --- | --- |
| Combined AEs | 7 (24.1%) | 4 (22.2%) | 1 (16.7%) | p=0.9 |
| Combined Biliary Recurrence | 5 (17.9%) | 5 (31.2%) | 0 (0%) | p=0.3 |
| *Kaplan-Meier analyses* |  |  |  |  |
| Mean estimated Dysfunction-free-Survival (95% CI), days | 475 (CI 294-656) | 195 (CI 104-287) | 209 (CI 209-209) | Log-rank p=0.4 |
| DFS Probability |  |  |  |  |
| 30 days | 100% | 100% | 100% |  |
| 3 months | 84% | 74% | 100% |  |
| 6 months | 72% | 41% | 100% |  |
| 1 year | 58% | 41% | 100% |  |
| Only technically and clinically successful combinations included.  TPS : Transpapillary Self-Expandable Metal Stent ; EUS-GE : EUS-guided Gastroenterostomy ; AEs : Adverse Events ; EUS-CD : EUS-guided Choledochoduodenostomy ; EUS-HG : EUS-guided Hepaticogastrostomy ; DFS : Dysfunction-Free Survival. | | | | |
